# Kinase Inhibitor Pulldown Assay Identifies a Chemotherapy Response Signature in Triple-negative Breast Cancer Based on Purine-binding Proteins

**DOI:** 10.1158/2767-9764.CRC-22-0501

**Published:** 2023-08-15

**Authors:** Junkai Wang, Alexander B. Saltzman, Eric J. Jaehnig, Jonathan T. Lei, Anna Malovannaya, Matthew V. Holt, Meggie N. Young, Mothaffar F. Rimawi, Foluso O. Ademuyiwa, Meenakshi Anurag, Beom-Jun Kim, Matthew J. Ellis

**Affiliations:** 1 Lester and Sue Smith Breast Center and Dan L. Duncan Comprehensive Cancer Center, Baylor College of Medicine, Houston, Texas.; 2 Department of Molecular and Cellular Biology, Baylor College of Medicine, Houston, Texas.; 3 Mass Spectrometry Proteomics Core, Advanced Technology Cores, Baylor College of Medicine, Houston, Texas.; 4 Department of Biochemistry and Molecular Biology, Baylor College of Medicine, Houston, Texas.; 5 Siteman Comprehensive Cancer Center and Washington University School of Medicine, St. Louis, Missouri.; 6 Department of Medicine, Baylor College of Medicine, Houston, Texas.; 7 AstraZeneca, Gaithersburg, Maryland.

## Abstract

Triple-negative breast cancer (TNBC) constitutes 10%–15% of all breast tumors. The current standard of care is multiagent chemotherapy, which is effective in only a subset of patients. The original objective of this study was to deploy a mass spectrometry (MS)-based kinase inhibitor pulldown assay (KIPA) to identify kinases elevated in non-pCR (pathologic complete response) cases for therapeutic targeting. Frozen optimal cutting temperature compound–embedded core needle biopsies were obtained from 43 patients with TNBC before docetaxel- and carboplatin-based neoadjuvant chemotherapy. KIPA was applied to the native tumor lysates that were extracted from samples with high tumor content. Seven percent of all identified proteins were kinases, and none were significantly associated with lack of pCR. However, among a large population of “off-target” purine-binding proteins (PBP) identified, seven were enriched in pCR-associated samples (*P* < 0.01). In orthogonal mRNA-based TNBC datasets, this seven-gene “PBP signature” was associated with chemotherapy sensitivity and favorable clinical outcomes. Functional annotation demonstrated IFN gamma response, nuclear import of DNA repair proteins, and cell death associations. Comparisons with standard tandem mass tagged–based discovery proteomics performed on the same samples demonstrated that KIPA-nominated pCR biomarkers were unique to the platform. KIPA is a novel biomarker discovery tool with unexpected utility for the identification of PBPs related to cytotoxic drug response. The PBP signature has the potential to contribute to clinical trials designed to either escalate or de-escalate therapy based on pCR probability.

**Significance:**

The identification of pretreatment predictive biomarkers for pCR in response to neoadjuvant chemotherapy would advance precision treatment for TNBC. To complement standard proteogenomic discovery profiling, a KIPA was deployed and unexpectedly identified a seven-member non-kinase PBP pCR-associated signature. Individual members served diverse pathways including IFN gamma response, nuclear import of DNA repair proteins, and cell death.

## Introduction

A total of 10% to 15% of breast tumors do not express estrogen receptor (ER) or progesterone receptor (PR), and are HER2 amplification negative (triple-negative breast cancer—TNBC; ref. 1). While TNBC is considered a single clinical entity and treated similarly, these tumors are highly diverse at both the clinical and molecular levels (1, 2). In the neoadjuvant chemotherapy treatment setting, approximately 40%–65% of patients have a pathologic complete response (pCR) in the breast and lymph nodes, depending on the chemotherapy regimen and whether programmed cell death 1 (PD1)/programmed death 1 ligand 1 (PD-L1)–directed antibodies are used (3, 4). When patients experience pCR, it is highly associated with improved overall survival (5, 6). Therefore, pCR is a valuable intermediate endpoint for designing new treatment regimens and triaging patients to salvage therapies, such as capecitabine or olaparib (7, 8). Unfortunately, there are currently no reliable methods to predict the likelihood of pCR from a pretreatment biopsy. An accurate pCR predictor would identify patients with a lower chance of response who can be better served by a clinical trial than an inadequate standard of care. For patients with a high chance of pCR, investigations could focus on developing lower toxicity regimens.

**Translational Relevance**

The identification of pretreatment predictive biomarkers for pathologic complete response (pCR) in response to neoadjuvant chemotherapy would advance precision treatment for triple-negative breast cancer (TNBC). To complement standard proteogenomic profiling, an optimized version of a kinase inhibitor pulldown assay (KIPA) was deployed in an analysis of 43 baseline TNBC biopsy samples collected before docetaxel and carboplatin neoadjuvant chemotherapy. While no kinase-based significant pCR associations emerged, seven non-kinase purine-binding proteins had positive associations with pCR, including GBP2, GBP5, RAC2, ATP6V1B2, NEDD4L, LDHB, and KPNA4. Validation using orthogonal mRNA datasets showed that a composite purine-binding protein signature predicted chemotherapy responsiveness and favorable TNBC outcomes. Significant correlations were also observed with IFN response pathways, immune scores, and immune checkpoint levels (PD-L1). Single-cell RNA sequencing identified GBP5 and RAC2 as T-cell associated. Thus, KIPA is a novel biomarker discovery tool with potential for defining immunoreactive and chemotherapy sensitive tumor types.

Mass spectrometry (MS)-based proteomics has successfully been employed to identify proteins and phosphoproteins differentially expressed in different breast cancer subtypes (9, 10). To increase the sensitivity and accuracy of profiling protein kinases as drug targets, multiplexed kinase inhibitor–conjugated beads (MIB) combined with quantitative MS have been used for biomarker discovery. For example, the MIB technique has been used to examine kinome reprogramming in leukemia and TNBC after treatment with kinase inhibitors (11, 12). Herein, we employ an optimized version of MIB called the kinase inhibitor pulldown assay (KIPA), based on nine kinase inhibitors relevant to both breast cancer treatment and kinase class coverage (13). The original intent of the project was to explore kinase expression in the setting of chemotherapy resistance to identify therapeutic targets to improve outcomes. However, during the investigation, unbiased data analysis revealed a much deeper source of potential biomarkers because of the large number of non-kinase purine-binding proteins (PBP) captured by the kinase inhibitor–conjugated bead approach.

This study was based on biospecimens collected from two previously published clinical trials (NCT02547987 and NCT02124902; ref. 14). Patients with clinical stages II/III TNBC received six cycles of docetaxel and carboplatin neoadjuvant chemotherapy before surgery (14). Microscaled proteogenomic profiling (15) was previously conducted on optimal cutting temperature compound (OCT)-embedded core needle biopsy samples collected before treatment from these two trials (16). KIPA was subsequently applied to the same sample set to identify additional predictive biomarkers for chemotherapy response.

## Materials and Methods

### Data Collection

Snap-frozen OCT-embedded core needle biopsies used in this study were part of the Clinical Proteomic Tumor Analysis Consortium (CPTAC)-TNBC study (14, 16), in which eligible patients including premenopausal or postmenopausal women ≥ 18 years old with clinical stages II/III ER-negative and HER2-negative (0 or 1+ by IHC or FISH negative) invasive breast cancer were recruited. The details of the clinical trials and proteogenomic studies can be found in previous publications (14, 16). The clinical trials were approved by the Institutional Review Board at both participating sites, WashU and BCM, and written informed consent from the patients was obtained. The studies were conducted in accordance with recognized ethical guidelines and followed the Declaration of Helsinki and Good Clinical Practice guidelines. In this study, 43 samples were successfully used for KIPA, which required a separate detergent-based native protein extraction approach (see below). The corresponding treatment response information (including pCR status and residual cancer burden class—RCB), molecular subtype information, RNA sequencing (RNA-seq), tandem mass tag (TMT)-based proteomics and phosphoproteomics data were obtained from the CPTAC-TNBC study (16). Protein-based and RNA-based immune score and multigene proliferation score (MGPS) were calculated as described previously (16). Programmed death 1 ligand 1 (PD-L1) levels from IHC staining were also described previously, as was the IHC protocol and pathology slide scoring method (16).

The primary validation dataset was derived from the BrighTNess phase III randomized TNBC clinical trial (17). Other datasets used for validation comprised of Silver and colleagues dataset (18), METABRIC (19), and TCGA datasets (20). The gene expression profiles of the BrighTNess dataset (GSE164458) and the Silver and colleagues dataset (GSE18864) was retrieved from the Gene Expression Omnibus (RRID:SCR_005012). Gene expression profiles and clinical outcome data of the METABRIC and TCGA datasets were accessed online using cBioPortal (RRID:SCR_014555).

### Kinobeads Preparation

Kinase inhibitors were conjugated with local laboratory generated ECH Sepharose 4B using the carbodiimide coupling method (12, 13). Briefly, nine kinase inhibitors (Palbociclib, Crizotinib, GSK690693, AZD4547, CZC-8004, Afatinib, FRAX597, Abemaciclib, and Axitinib; Supplementary Table S1) were separately conjugated to homemade ECH Sepharose 4B via carbodiimide coupling chemistry as described previously (12). ECH Sepharose 4B was synthesized using conjugating 6-Aminohexanoic acid (Sigma) to cyanogen bromide (CNBr)-activated Sepharose 4B (GE Healthcare). Each kinase inhibitor was dissolved in 50% dimethylformamide (DMF)/ethanol (EtOH) and added to the ECH Sepharose 4B beads in the presence of 0.1 mol/L 1-ethyl-3-(3-dimethylaminopropyl)carbodiimide and allowed to react overnight at 4°C with rotation. After coupling, the unreacted groups were inactivated with ethanolamine. Subsequently, beads were washed with 0.1 mol/L Tris-HCl, pH 8.3 with 500 mmol/L NaCl and 0.1 mol/L acetate, pH 4.0 with 500 mmol/L NaCl and stored in 20% ethanol at 4°C in the dark.

### Kinase Enrichment by Kinobeads Precipitation

Native protein lysates were extracted as described previously (15). OCT-embedded biopsy samples were washed with PBS three times and 100 μL of native protein lysis buffer [50 mmol/L HEPES (pH 7.5), 150 mmol/L NaCl, 0.5% Triton X-100, 1 mmol/L Ethylenediaminetetraacetic acid (EDTA), 1 mmol/L ethylene glycol-bis(β-aminoethyl ether)-N,N,N′,N′-tetraacetic acid (EGTA), 10 mmol/L NaF, 2.5 mmol/L Na_3_VO_4_, Protease inhibitor cocktail, Phosphatase inhibitor cocktail] was added to the samples. Samples were incubated on ice for 10 minutes and sonicated by the S220 Ultrasonicator for 2 minutes (Covaris). Protein concentration was measured by Protein Assay (Bio-Rad).

KIPA was performed as described previously (13). For each KIPA pulldown, 50 μg of native protein lysate was mixed with 10 μL of kinobeads previously equilibrated in lysis buffer for 1 hour at 4°C with rotation. Kinobeads and their bound proteins were pulled down by centrifugation at 600 × *g* for 30 seconds, and the supernatant containing unbound proteins was aspirated. The beads were washed twice with 400 μL buffer containing 50 mmol/L HEPES (pH 7.5), 600 mmol/L NaCl, 1 mmol/L EDTA, 1 mmol/L EGTA with 0.5% Triton X-100, and twice with the same buffer without Triton X-100 followed by two washes with water. After final wash, bound proteins were digested with trypsin overnight at 37°C. To remove the remaining detergent from the lysis buffer prior to MS analysis, the digested peptide mixture was processed using a HiPPR Detergent Removal Kit (Thermo Fisher Scientific) according to the manufacturer's instructions and dried using a speed-vac prior to MS analysis.

### Proteomic Data Acquisition and Processing

KIPA samples were analyzed by MS as described previously (15). Peptides generated from KIPA were analyzed using an Orbitrap Fusion Lumos mass spectrometer coupled with an EASY-nLC1200 system (Thermo Fisher Scientific). One-third of the KIPA sample was loaded onto a trap column (150 μm × 2 cm, particle size 1.9 μm) with a maximum pressure of 280 bar using Solvent A (0.1% formic acid in water) and then separated on a silica microcolumn (150 μm × 5 cm, particle size, 1.9 μm) with a gradient of 4%–28% mobile phase B (90% acetonitrile and 0.1% formic acid) at a flow rate of 750 nL per minute for 75 minutes.

The data-dependent acquisition (DDA) mode was used. For DDA, a precursor scan between m/z 300 to 1,200 was performed in the Orbitrap at 120,000 resolution at 200 m/z. The 20 most intense ions were isolated by quadrupole with a 2 m/z window, and fragmented by higher energy collisional dissociation with a normalized collision energy of 32%, and detected by ion trap with a rapid scan rate. The automatic gain control targets were 5 × 10^5^ ions with a maximum injection time of 50 ms for precursor scans and 10^4^ ions with a maximum injection time of 50 ms for MS2 scans. The dynamic exclusion time was 20 seconds (±7 ppm). For relative quantification, the raw spectra were converted to the mgf format using Proteome Discoverer 2.0 software (Thermo Fisher Scientific, RRID:SCR_014477) and then imported to Skyline (21) along with the raw data file.

### Identification of Differential Proteins from the KIPA

Differential proteins were identified with the KIPA data using Wilcoxon rank-sum tests. Missing values (NA) were imputed as 0 during analysis. Proteins with log_10_(fold change) > 1 and *P* value < 0.05 were taken as differential proteins between pCR and non-pCR samples. The PBP signature score was calculated as the average level of seven signature genes (GBP2, GBP5, RAC2, ATP6V1B2, NEDD4L, LDHB, and KPNA4) measured by different platforms (KIPA, proteomics, or RNA-seq).

The number of publications corresponding to each signature protein was performed using R package RISmed (22). The peer-reviewed publications were extracted from the PubMed database, and each gene symbol was searched with the keywords “breast cancer” and “chemotherapy” to find relevant publications. These tasks were performed in an in-house R script, where the final query for this publication was performed on April 3, 2023.

R package “ggplot2” (23) and “ComplexHeatmap” (24) were applied to generate volcano plots and heat maps, respectively, to visualize the identified differential proteins.

### Overrepresentation Analysis and Gene Set Enrichment Analysis

Total 2,641 proteins detected by the KIPA data were subjected to Gene Ontology (GO) overrepresentation analysis (ORA) to identify enriched molecular function (MF) terms using the R package “WebGestaltR” (25).

Gene set enrichment analysis (GSEA, RRID:SCR_003199) was performed using publicly available software (GSEA version 4.2; ref. 26). The input of GSEA is a ranked list of signed −log_10_*P* values from Pearson correlation between the KIPA-based PBP signature score and the genes measured by either the proteomics or RNA-seq in the CPTAC-TNBC dataset. The Hallmark gene sets within the Molecular Signatures Database (MSigDB) were used for GSEA. A FDR of 0.05 was utilized to define the statistical significance of GSEA in this study.

### Statistical and Validation Analysis

The protein and mRNA levels of seven signature genes were compared with a correlation matrix using the Spearman method. A correlation matrix was generated using the R package “corrplot” (27).

ROC curve analysis was applied to RNA-seq data of tumor samples from the BrighTNess dataset. The R package “pROC” (28) was used to draw the ROC curves and calculate the AUC.

Survival analysis was performed using the R package “survival” (29) to evaluate the prognostic effects of the prioritized genes. Disease-specific survival was defined as the time from the date of diagnosis to the date of death due to breast cancer. The Cox proportional hazards regression model (Wald test) was used to calculate univariate and multivariate hazard ratios.

The expression patterns of seven signature genes in different annotated cell types were generated from a public single-cell RNA-seq dataset (30). The figures were generated from the Broad Institute Single Cell portal at https://singlecell.broadinstitute.org/single_cell/study/SCP1039.

### Data Availability

The KIPA data generated in this study are available upon request. The data used for validation purposes in this study were obtained from Gene Expression Omnibus (RRID:SCR_005012) at GSE164458 and GSE18864.

## Results

### Sample Overview and KIPA Workflow

**FIGURE 1:**
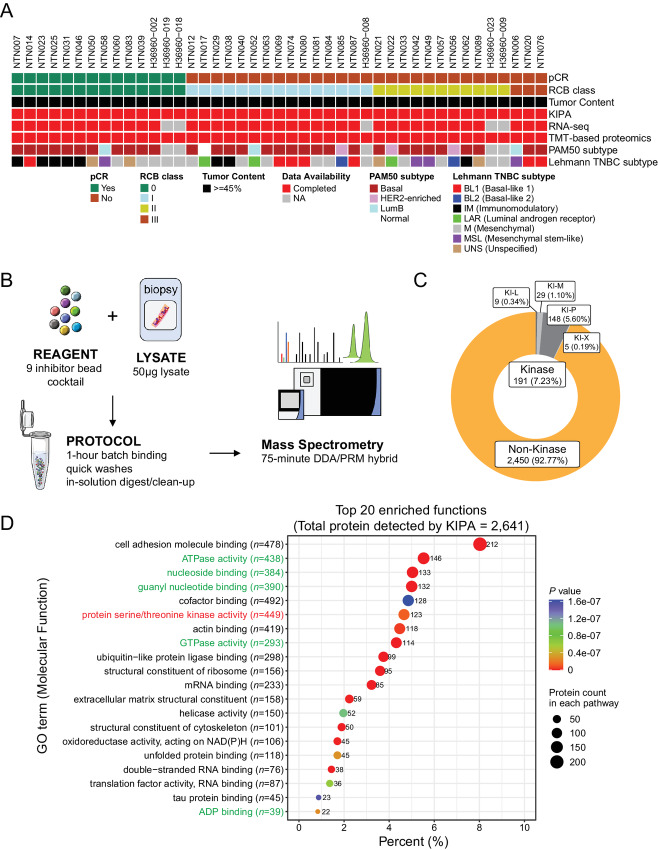
The application of the KIPA in the CPTAC-TNBC samples. **A,** Overview of the 43 pretreatment biopsy samples used in this study (14 pCR samples and 29 non-pCR samples). The biopsy samples were accrued from patients with TNBC enrolled in two clinical trials (NCT02547987 and NCT02124902) who were treated with six cycles of docetaxel and carboplatin (14). pCR, RCB class, omics data availability, and molecular subtypes were obtained from previous publication (16) and are indicated via color-coded annotation tracks. **B,** The workflow of KIPA was similar as described previously (13). Native lysates from OCT-embedded biopsy samples were incubated with homemade kinobeads (9 inhibitor bead cocktail) for 1 hour, followed by multiple washing steps and in-solution digestion. Quantitative MS was used for accurate identification and quantification. **C,** Protein classes of total 2,641 proteins detected by KIPA in 43 biopsy samples. KI-L, lipid kinase; KI-P, protein kinase, KI-M, metabolic kinase, KI-X, uncategorized kinase. **D,** Top 20 molecular function terms of GO ORA with a total of 2,641 proteins detected by KIPA. The color of each dot represents the significance (*P*-value) of enrichment, and the size is proportional to the overlapping number between 2,641 proteins and each term (also indicated by the labeled number). The red text denotes kinase-associated function while green denotes nucleotide-associated functions.

The CPTAC-TNBC study collected snap-frozen OCT-embedded core needle biopsies from consented patients with clinical stages II/III TNBC (NCT02547987 and NCT02124902; refs. 14, 16). The patients were treated with six cycles of neoadjuvant docetaxel and carboplatin combination chemotherapy (14, 16). The previously described BioTExt (Biopsy Trifecta Extraction) method (15) was used to process the samples and profile the proteogenomic landscape of the biopsy samples (16). In addition to the proteins detected by the microscaled proteogenomic methods, KIPA was developed to specifically profile the human kinome and associated proteins from clinical tumor lysates (13). To identify kinases and kinase-associated proteins whose abundance can predict neoadjuvant chemotherapy response, KIPAs were performed using 43 baseline samples (collected before treatment) from the CPTAC-TNBC study (Supplementary Tables S2 and S3). Homemade kinobeads containing nine kinase inhibitor–conjugated beads (Palbociclib, Crizotinib, GSK690693, AZD4547, CZC-8004, Afatinib, FRAX597, Abemaciclib, and Axitinib) were used to capture the critical druggable kinases and their binding proteins. Enriched proteins were identified and quantified using quantitative MS for further analysis (Fig. 1A and B).

From the 43 samples, KIPA identified a total of 2,641 proteins, most of which were also identified by the RNA-seq and global proteomics of the same samples (Supplementary Fig. S1A). A total of 191 of 2,641 proteins identified (7.23%) belong to the human kinase family (Fig. 1C). Most of the proteins detected (2,450, 92.77%) were classified as non-kinase proteins (Fig. 1C; Supplementary Table S4). This finding is not surprising because it has been reported that in addition to kinases, kinobeads can also bind some ATPs and PBPs, such as chaperones, helicases, adenosine triphosphatases (ATPase), and guanosine triphosphatase (GTPase; ref. 31). To better understand the biological functions of all the proteins identified, 2,641 proteins were subjected to GO ORA (Fig. 1D). In addition to protein serine/threonine kinases, proteins were enriched in MF terms including ATPase activity and nucleoside/guanyl binding (Fig. 1D). Hence, kinase inhibitor–conjugated beads could capture not only targetable human kinases, but also many non-kinases that either bind to kinases or directly bind to kinobeads. We therefore focused on the prognostic implications of all the proteins and aimed to prioritize robust biomarkers of chemotherapy response.

### Identification of PBP Signature Associated with pCR Samples

**FIGURE 2:**
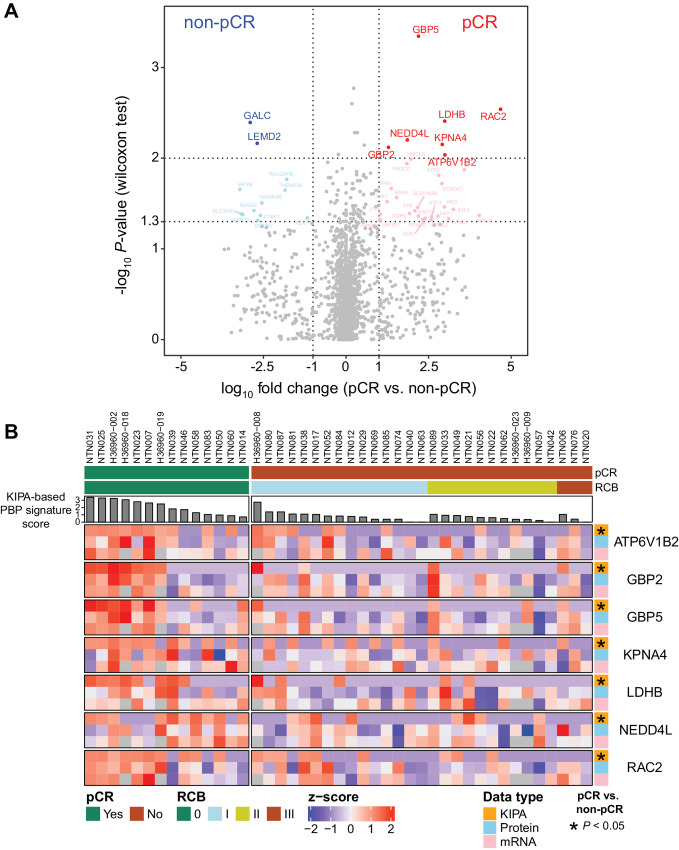
Differential protein analysis between pCR and non-pCR samples. **A,** Volcano plot shows results from differential analysis between pCR and non-pCR samples with the KIPA. *X*-axis is the log_10_ fold change between median value of pCR and non-pCR samples for each protein. *Y*-axis is the −log_10_*P*-value from Wilcoxon rank-sum tests comparing protein levels in pCR samples to non-pCR samples. Red and blue points indicate the most significant (*P* < 0.01) proteins, and pink and light blue points indicate significant (0.01 < *P* < 0.05) proteins. **B,** Heat map shows the levels of seven PBP signature genes across 43 samples, arranged by RCB class (0, I, II, II) and the KIPA-based PBP signature score. The levels of seven genes were assessed at the level of KIPA (orange), protein (light blue), and mRNA (pink), and normalized across all samples by z-score. *P* values were determined by Wilcoxon rank-sum tests comparing gene levels for pCR samples to non-pCR samples. PBP signature, purine-binding protein signature.

To identify predictive biomarkers for neoadjuvant chemotherapy response, Wilcoxon rank-sum tests were performed on the KIPA data (Supplementary Table S5). Only four kinases were identified to be upregulated in pCR samples and there were no kinases upregulated in non-pCR samples (*P* < 0.05; Supplementary Fig. S1B). Among all the proteins detected by KIPA, 43 differential proteins were identified (*P* < 0.05; Fig. 2A). A total of 31 proteins were significantly upregulated and 12 proteins were downregulated in pCR samples (Fig. 2A). Examples such as GALC (Galactosylceramidase) and LEMD2 (LEM domain nuclear envelope protein 2) were significantly higher in non-pCR samples. In contrast, GBP5 (guanylate binding protein 5), which belongs to the GTPase family, was the most significantly elevated protein in pCR samples (Fig. 2A). Compared with the proteogenomics study previously applied to the same set of biopsy samples (16), most differential proteins from the KIPA analysis were not identified as significant (*P* < 0.05) by either TMT-based global proteomics or RNA-seq data, although the trends for most gene products were consistent (Supplementary Fig. S2A). This finding suggests that besides standard proteogenomic profiling, our kinase inhibitor bead-based enrichment method provides a useful orthogonal platform for biomarker discovery studies.

To identify a protein signature associated with chemotherapy sensitivity, the most significantly upregulated proteins in pCR samples were prioritized [*P* < 0.01, including GBP2, GBP5, RAC2 (Rac Family Small GTPase 2), ATP6V1B2, NEDD4L, LDHB, and KPNA4]. The signature includes seven non-kinase proteins, among which GBP2, GBP5, and RAC2 are GTPases and bind to GTP. ATP6V1B2 (ATPase H+ Transporting V1 Subunit B2) is a component of the vacuolar ATPase enzyme complex and binds to ATP (32). NEDD4 L (NEDD4 like E3 ubiquitin protein ligase) is an E3 ubiquitin ligase and binds to ATP through the process of ubiquitination (33). LDHB (lactate dehydrogenase B) is an enzyme that catalyzes the conversion of pyruvate to lactate, during which LDHB binds to NADH and NAD+ (34). KPNA4 (Karyopherin subunit α4, or importin-α3) forms a heterodimer with importin β (KPNB1) and is responsible for nuclear transportation of nuclear localization signals (NLS)-containing proteins (35). After nuclear transportation, RAN (a GTPase) rapidly binds to the importin β to dissociate the complex, releasing the transported protein and recycling of the importins back to the cytoplasm (35). Therefore, KPNA4 indirectly binds GTP through the RAN GTPase. Because GTP, ATP, and NAD+ are all purine-based nucleotides, we termed this seven-member signature PBP signature. To investigate the novelty of this PBP signature, the number of publications corresponding to each signature protein related to chemotherapy treatments in breast cancer was obtained. The peer-reviewed publications were extracted from the PubMed database, and each gene symbol was searched with the keywords “chemotherapy” and “breast cancer” using R package RISmed. LDHB has five, NEDDL4 has two, GBP5, GBP2, and RAC2 have one citation each, while KPNA4 and ATP6V1B2 have no citations (Supplementary Fig. S2B).

The KIPA-based PBP signature score, which was derived from the average level of seven proteins measured by the KIPA, showed a significant enrichment in pCR samples and a partially concordant pattern with the protein and mRNA expression levels of each individual gene (Fig. 2B). Despite the positive correlations observed between protein and mRNA levels for seven signature genes (Supplementary Fig. S2C), KIPA-based PBP signature scores were the most significantly enriched in pCR samples (Wilcoxon test *P* = 5.5e-5) compared with protein-based PBP signature scores (Wilcoxon test *P* = 0.07) and mRNA-based PBP signature scores (Wilcoxon test *P* = 0.04; Supplementary Fig. S3).

**FIGURE 3:**
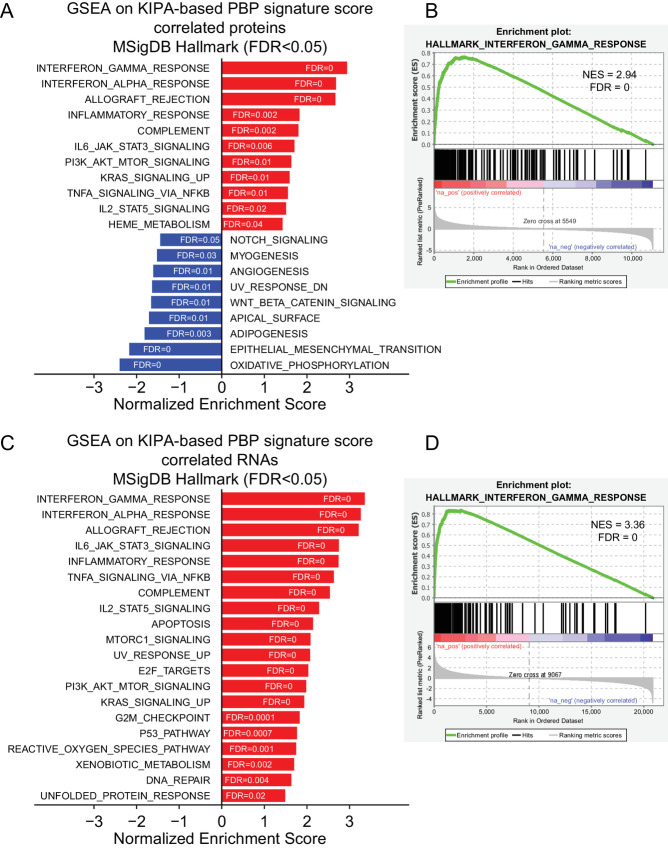
GSEA of the PBP signature-correlated genes. **A,** Bar plot shows the enriched Hallmark gene sets (FDR < 0.05) from GSEA of the KIPA-based PBP signature score-correlated genes at the protein levels. GSEA input was a ranked list of signed −log_10_*P*-values from Pearson correlation between the KIPA-based PBP signature score and all the genes measured by the global proteomics in the CPTAC-TNBC dataset. Pathways with positive NES (normalized enrichment score) were positively correlated with the KIPA-based PBP signature score, while pathways with negative NES were negatively correlated. **B,** Enrichment plots for INTERFERON_GAMMA_RESPONSE Hallmark pathway at the protein levels. **C,** Bar plot shows the enriched Hallmark gene sets (FDR < 0.05) from GSEA of the KIPA-based PBP signature score-correlated genes at the mRNA levels. GSEA input was a ranked list of signed −log_10_*P*-values from Pearson correlation between the KIPA-based PBP signature score and all the genes measured by the RNA-seq. **D,** Enrichment plots for INTERFERON_GAMMA_RESPONSE Hallmark pathway at the mRNA levels.

To explore the pathways that were associated with the PBP signature score, GSEA was performed using a ranked list of signed −log_10_*P* values from Pearson correlation between the KIPA-based PBP signature scores and the expression levels of all the genes (Fig. 3A--D). The significantly enriched protein-based and mRNA-based pathways within the Hallmark gene sets were shown (FDR < 0.05; Fig. 3A and C). At both protein and mRNA levels, IFN gamma response pathway is the most positively correlated pathway with the KIPA-based PBP signature score (FDR = 0; Fig. 3A–D). The enrichment plots for IFN gamma response pathway are shown in Fig. 3B and D. The leading-edge genes from the Hallmark IFN gamma pathway also showed an enrichment in pCR samples (Supplementary Fig. S4).

### Validation of the PBP Signature in Predicting Neoadjuvant Chemotherapy in TNBC

**FIGURE 4:**
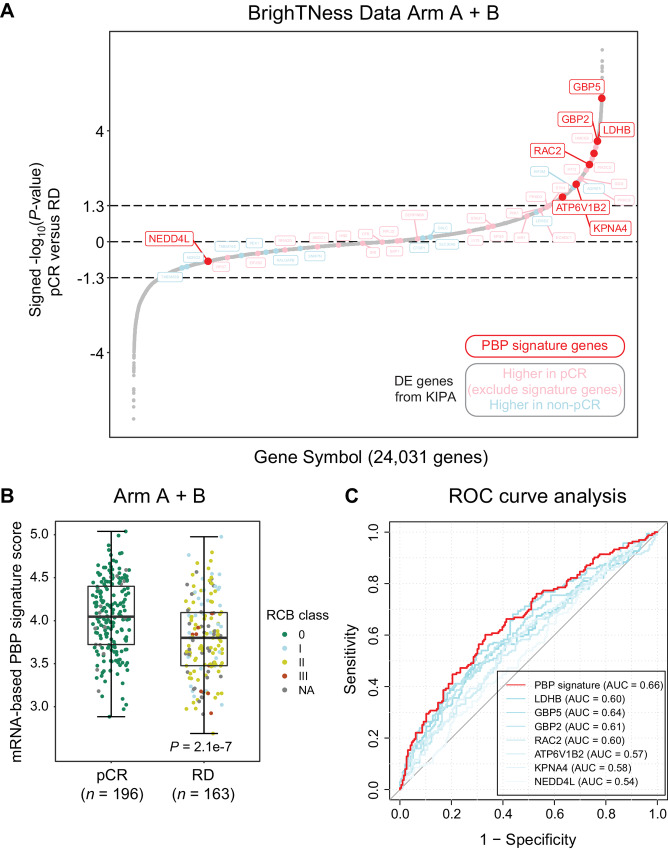
Validation of candidates identified from the KIPA analysis in the BrighTNess dataset. **A,** The differential proteins from the KIPA analysis were examined in the BrighTNess dataset (Arm A + B) where patients with TNBC were treated with carboplatin and paclitaxel with or without veliparib. The 24,031 genes were ranked on the basis of signed −log_10_ of *P* values from Wilcoxon rank-sum tests comparing mRNA levels for pCR samples with RD samples. Positive values indicate higher expression in pCR samples, and negative values indicate higher expression in RD samples. The PBP signature genes were indicated as red dots, and other differential proteins from the KIPA analysis were indicated as either pink (higher in pCR samples) or light blue (higher in non-pCR samples) dots. **B,** The levels of mRNA-based PBP signature in pCR and RD samples. Boxplots show interquartile range (IQR) with median marked in center. Whiskers indicate 1.5x IQR. *P* value was determined by Wilcoxon rank-sum test between pCR and RD samples. **C,** ROC curve analysis to test the validity of mRNA-based PBP signature score and mRNA levels of individual signature genes in discriminating pCR and RD samples. ROC, receiver operating characteristic; AUC, area under the ROC curve.

Independent datasets were deployed to validate the performance of the PBP signature identified from the KIPA analysis in predicting chemotherapy response of patients with TNBC, particularly those who received the regimen of taxanes and carboplatin combination. The BrighTNess cohort is a phase III, randomized, double-blind, placebo-controlled trial that recruited patients with stage II/III TNBC (17). Patients were randomized to receive three different neoadjuvant regimens: paclitaxel plus carboplatin plus veliparib (Arm A), paclitaxel plus carboplatin (Arm B), or paclitaxel only (Arm C). The tumor samples were collected before the neoadjuvant treatment and RNA-seq analysis was performed for each sample (36). The addition of veliparib in Arm A did not increase the proportion of patients who achieved pCR (17). Therefore, Arm A and Arm B were combined (*n* = 359) to investigate the potential biomarkers initially identified from the KIPA analysis. By performing Wilcoxon rank-sum tests between pCR and residual disease (RD) groups, all 24,031 genes were ranked on the basis of the signed −log_10_*P*-value (Fig. 4A). A positive value of a certain gene indicates its upregulated expression in pCR samples, whereas a negative value represents downregulation in pCR samples.

A total of 42 of the 43 differential genes from the KIPA analysis were also detected by RNA-seq of the BrighTNess dataset and are shown as colored dots in Fig. 4A. Six of seven PBP signature genes were significantly upregulated in pCR samples in the BrighTNess dataset (*P* < 0.05; Fig. 4A). Among seven signature genes, GBP5 was the most significantly upregulated genes in pCR samples and ranked 22 of all 24,031 examined genes (Fig. 4A). Although six of seven signature genes show a significant increase in pCR samples (Supplementary Fig. S5A), the enrichment of mRNA-based PBP signature score (derived from the average mRNA level of seven genes) in pCR samples was the most significant (Wilcoxon test *P* = 2.1e-7; Fig. 4B). ROC curve analysis revealed that the area under the ROC curve (AUC) for the PBP signature was 0.66, outperforming any individual signature gene (Fig. 4C). This finding indicates that at the mRNA level, the PBP signature is a modest biomarker for predicting chemotherapy response in the BrighTNess mRNA dataset.

Another clinical trial where a modest number of patients with TNBC (*n* = 24) were treated with single-agent cisplatin neoadjuvant therapy was also used as a validation set (18). The mRNA-based PBP signature scores significantly increased with the increasing clinical responses (i.e., increases from progressive disease to stable disease, to partial response, to complete response; Jonckheere–Terpstra test *P* = 0.02; Supplementary Fig. S5B).

### Evaluation of Prognostic Effects of Differential Proteins in TNBC Subtype

To investigate the long-term effects of predictive biomarkers on the prognosis of patients with TNBC, the METABRIC dataset was used (19). The METABRIC dataset consists of nearly 2,000 breast cancer samples with a median 10-year follow-up, detailed clinical information, and genome-wide gene expression data (19). Basal-like and claudin-low breast cancer samples (*n* = 398) were used to evaluate the prognostic effects of the PBP signature from the KIPA analysis. A total of 398 samples were stratified on the basis of the median expression of each gene and survival analysis was performed to obtain the *P* value and HR. HR > 1 indicates that patients with higher expression of a certain gene have a worse prognosis, and HR < 1 indicates a better prognosis.

**FIGURE 5:**
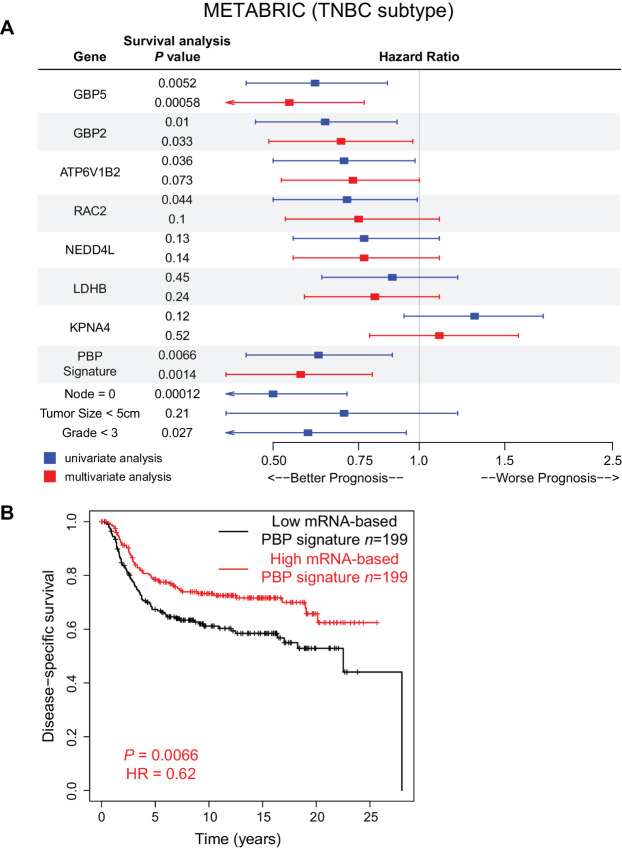
The prognosis effects of the KIPA-based PBP signature in METABRIC dataset. **A,** Forest plot shows HRs and *P* values for disease-specific survival associated with the PBP signature and seven signature genes. HR and 95% confidence intervals were based on categorizing TNBC samples using a median mRNA expression cutoff for each gene or the PBP signature score in the METABRIC dataset. Survival analysis was also performed for clinical factors (lymph nodes number, tumor size, and tumor grade). Blue boxes indicate univariate analysis, and red boxes indicate multivariate analysis. **B,** Kaplan–Meier curve shows disease-specific survival of TNBC samples from the METABRIC dataset. Samples were categorized into mRNA-based PBP signature score high and low group based on the median cutoff.

In the univariate analysis, four of seven signature genes (GBP5, RAC2, GBP2, and ATP6V1B2) showed significant prognostic effects (*P* < 0.05; Fig. 5A; Supplementary Fig. S6). The multivariate survival analysis demonstrated that higher mRNA levels of GBP5 and GBP2 were significantly correlated with better disease-specific survival (Fig. 5A). Interestingly, the higher mRNA-based PBP signature scores were associated with favorable clinical outcomes from both univariate and multivariate analysis (Fig. 5A and B). In addition, in TCGA pan-cancer dataset, although the PBP signature overall was not prognostic, the higher expression levels of individual signature gene members, such as GBP5 and ATP6V1B2, were associated with better disease-specific survival (Supplementary Fig. S7).

Combined with previous validation with the BrighTNess and the Silver and colleagues dataset (Fig. 4; Supplementary Fig. S5), we concluded that high mRNA-based PBP signature scores in TNBC samples were associated with chemotherapy response and favorable clinical outcomes.

### Relationship Between the PBP Signature Scores, Immune Scores, and PD-L1 Levels

**FIGURE 6:**
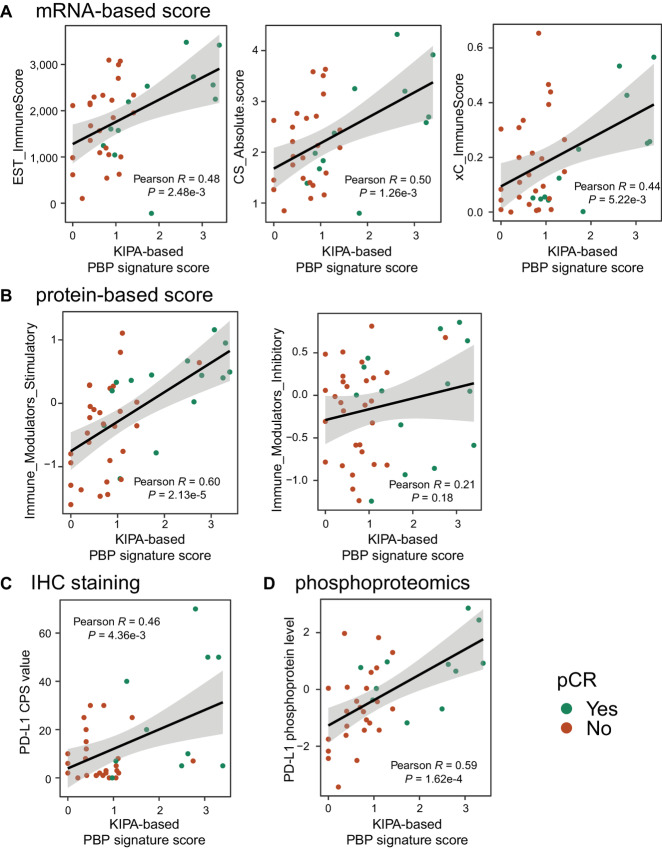
The relationship between the KIPA-based PBP signature score, immune scores, and immune checkpoint levels. **A,** Scatter plot shows the correlation between the KIPA-based PBP signature score and mRNA-based immune scores (from ESTIMATE, Cibersort, and xCell). EST: ESTIMATE, CS: Cibersort, xC: xCell. **B,** Scatter plot shows the correlation between the KIPA-based PBP signature score and protein-based immune modulator stimulatory or inhibitory scores. **C,** Scatter plot shows the correlation between the KIPA-based PBP signature score and PD-L1 CPSs from IHC staining. **D,** Scatter plot shows the correlation between the KIPA-based PBP signature score and PD-L1 phosphoprotein levels.

Because the PBP signature scores were highly correlated with the IFN gamma response pathway, the association between the PBP signature and immune scores derived from proteomics and RNA-seq data was further investigated with the CPTAC-TNBC dataset (Fig. 6). The KIPA-based PBP signature scores were positively correlated with mRNA-based immune scores derived from ESTIMATE (Pearson *R* = 0.48, *P* = 2.48e-3), Cibersort (Pearson *R* = 0.50, *P* = 1.26e-3), and xCell (Pearson *R* = 0.44, *P* = 5.22e-3; Fig. 6A). In addition, the KIPA-based PBP signature scores were highly positively correlated with protein-derived immune stimulatory scores (Pearson *R* = 0.60, *P* = 2.13e-5), but not with immune inhibitory scores (Pearson *R* = 0.21, *P* = 0.18; Fig. 6B). In contrast, there were no significant correlations between the signature levels and MGPSs, indicating a low connection between the PBP signature and tumor proliferation pathways (Supplementary Fig. S8A).

IHC staining of tumor sample slides revealed a combined positive score (CPS) of PD-L1 for each sample. A significant positive correlation between PD-L1 CPS values and the KIPA-based PBP signature scores was observed (Pearson *R* = 0.46, *P* = 4.36e-3; Fig. 6C). Similar correlations were observed between the PBP signature scores and PD-L1 phosphoprotein levels measured by phosphoproteomics (Pearson *R* = 0.59, *P* = 1.62e-4; Fig. 6D). These findings imply a potential connection to the potential benefits of immune checkpoint inhibitor treatment recently introduced for patients with TNBC. Combined with single-cell RNA-seq data previously conducted in 26 primary breast tumors (30), seven PBP signature genes show distinct expression patterns in different annotated cell types (Supplementary Fig. S8B and S8C). While some genes such as GBP5 and RAC2 are predominantly expressed in immune cells (T cells and myeloid cells), other signature genes exhibited universal expression in both tumor cells and immune or stromal compartments (Supplementary Fig. S8C).

## Discussion

Here, we describe the results from our recently established KIPA approach in clinical tumor samples to investigate predictive biomarkers for neoadjuvant chemotherapy response in TNBC. KIPA identified more than 300 human kinases by deploying a cocktail of nine experimental and FDA-approved kinase inhibitors conjugated to beads for enrichment, followed by quantitative MS (13). The intended targets of each kinase inhibitor were successfully captured and quantified in our previous experiments (13). The genesis of this article is that the KIPA pulldown method also detected many non-kinase proteins, most commonly purine-binding proteins such as ATPases and GTPases. Biomarker discovery analysis was performed by comparing tumors collected from patients who achieved a pCR after neoadjuvant docetaxel- and carboplatin-based chemotherapy to those who did not (14). While the objective of discovering druggable kinases in chemotherapy-resistant non–pCR-associated tumors was not met, the KIPA platform generated unexpected relationships between a spectrum of other non-kinase PBPs and pCR. These chemotherapy response-associated proteins prioritized by the KIPA platform might not be discovered using standard proteogenomic tools (TMT-based global proteomics and RNA-seq), either because KIPA allowed detection and quantification of low-abundance purine-binding proteins by enrichment or because the relationships with pCR occurred at the posttranscriptional level and therefore not detectable by RNA-seq. For example, the very weak validation of KPNA4 in RNA-seq data may be because its role in DNA repair is posttranscriptional, and ultimately protein-level validation will be required (37).

Among all protein candidates, a pCR-associated purine-binding protein signature was the core discovery described herein. This signature includes six PBPs (GBP2, GBP5, RAC2, ATP6V1B2, NEDD4L, and LDHB) and KPNA4 that likely binds to the KIPA beads indirectly via the RAN GTPase. Some of the signature proteins have already been reported to be associated with chemotherapy sensitivity, including GBP2 (38), GBP5 (39), RAC2 (40), and LDHB (41). The roles of the other three PBP signature proteins in predicting chemotherapy response have yet to be investigated in breast cancer, but a role is highly plausible. For example, KPNA4 (importin α3) is a subunit of the karyopherin nuclear transportation machinery and forms the importin α/β heterodimer (35). The multiple other karyopherin complex components, including KPNB1, KPNA2, KPNA3, and RAN, were also detected by KIPA in our study (Supplementary Table S3). Mechanistically, a recent MS study has reported that KPNA4 assists the translocation of cytoplasmic transcription factor CRIP1 into the nucleus along with the nuclear import of the BRCA2-RAD51 complex upon DNA damage (37). In contrast, ATP6V1B2 may play a role in cell death in response to chemotherapy. V-ATPases maintain pH homeostasis through lysosomal acidification and modulate autophagy, cell invasion, and cell death (42).

GSEA indicated that the PBP signature correlated most strongly with IFN gamma response–associated proteins. It is well established from previous publications that the IFN gamma pathway is elevated in pCR-associated samples (16). GBP2 and GBP5 are IFN-inducible GTPases involved in a broad spectrum of innate immune functions against intracellular pathogens (43, 44). RAC2 activates Th1-specific signaling and IFN gamma gene expression (45). NEDD4 L has been reported to promote type I IFN production in response to the virus by catalyzing ubiquitination of the cysteines in TRAF3 (TNF receptor associated factor 3; ref. 46). Single-cell RNA-seq analysis from a publicly available database (30) showed that some of the PBP signature genes are predominantly expressed in immune cells (GBP5 and RAC2), while other genes show universal expression patterns across cell types. Thus, we hypothesize that the elevated levels of the PBP signature in pCR samples are driven by both the cancer cells and the immune or stromal compartments.

Compared with other breast cancer subtypes, TNBC has the highest incidence of patients with tumor-infiltrating lymphocytes (TIL; ref. 47). After patients with TNBC receive neoadjuvant chemotherapy, the presence of TILs is associated with pCR, improved disease-free and overall survival (48, 49). PD-L1 is expressed in approximately 30% of patients with breast cancer, and its expression is positively associated with triple-negative status and high levels of TILs (50). Recent promising results of the KEYNOTE-522 trial led to the approval of the programmed cell death protein 1 (PD1)-targeting antibody pembrolizumab for neoadjuvant TNBC treatment (3). Pembrolizumab in combination with chemotherapy significantly increased event-free survival compared with chemotherapy alone (5). The high correlation between the PBP signature levels and the PD-L1 IHC and phosphoprotein levels implies the broader role of the PBP signature in predicting not just chemotherapy sensitivity but also immunotherapy sensitivity in TNBC. These findings will be worth testing in prospective TNBC cohorts where patients receive the current standard-of-care of chemotherapy plus immune checkpoint antibodies to further validate the approach.

In conclusion, low sample requirements (<50 μg protein lysate) and the short processing time (complete within 2 days) make KIPA highly applicable for efficient clinical investigation (13). KIPA reveals an unexpectedly rich source of PBPs as chemotherapy response biomarkers for distinguishing TNBC tumors that will or will not respond to chemotherapy. For patients with low PBP signature scores and therefore low pCR probability, treatment escalation with investigational drugs may be appropriate. For patients with high scores, treatment de-escalation could be considered. For example, in the presence of an immune checkpoint inhibitor, it may be possible to reduce the number of chemotherapy agents used to treat TNBC, which currently includes doxorubicin, cyclophosphamide, carboplatin, and a taxane.

## Supplementary Material

Supplementary Table S1-S5 Supplementary Table S1-S5

Supplementary Figure 1 Supplementary Figure 1

Supplementary Figure 2 Supplementary Figure 2

Supplementary Figure 3 Supplementary Figure 3

Supplementary Figure 4 Supplementary Figure 4

Supplementary Figure 5 Supplementary Figure 5

Supplementary Figure 6 Supplementary Figure 6

Supplementary Figure 7 Supplementary Figure 7

Supplementary Figure 8 Supplementary Figure 8
