# Supplementary material for: Kinase Inhibitor Pulldown Assay Identifies a Chemotherapy Response Signature in Triple-negative Breast Cancer Based on Purine-binding Proteins
Source: Cancer Res Commun. 2023 Aug 15;3(8):1551–63. doi: 10.1158/2767-9764.CRC-22-0501 (PMC10426551; doi:10.1158/2767-9764.CRC-22-0501)
Supplement: Supplementary Figure 1 [file crc-22-0501-s02.pdf]

Supplementary Figure 1

A

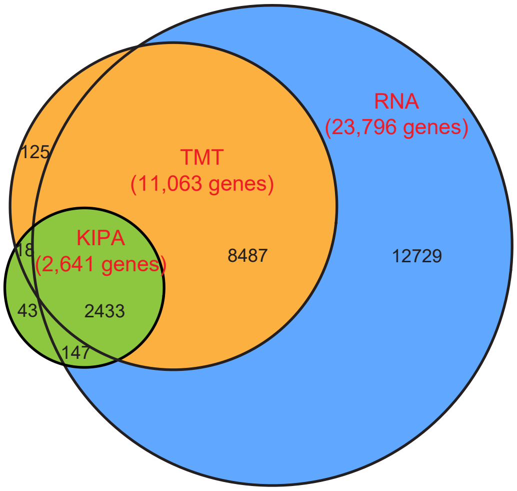

B

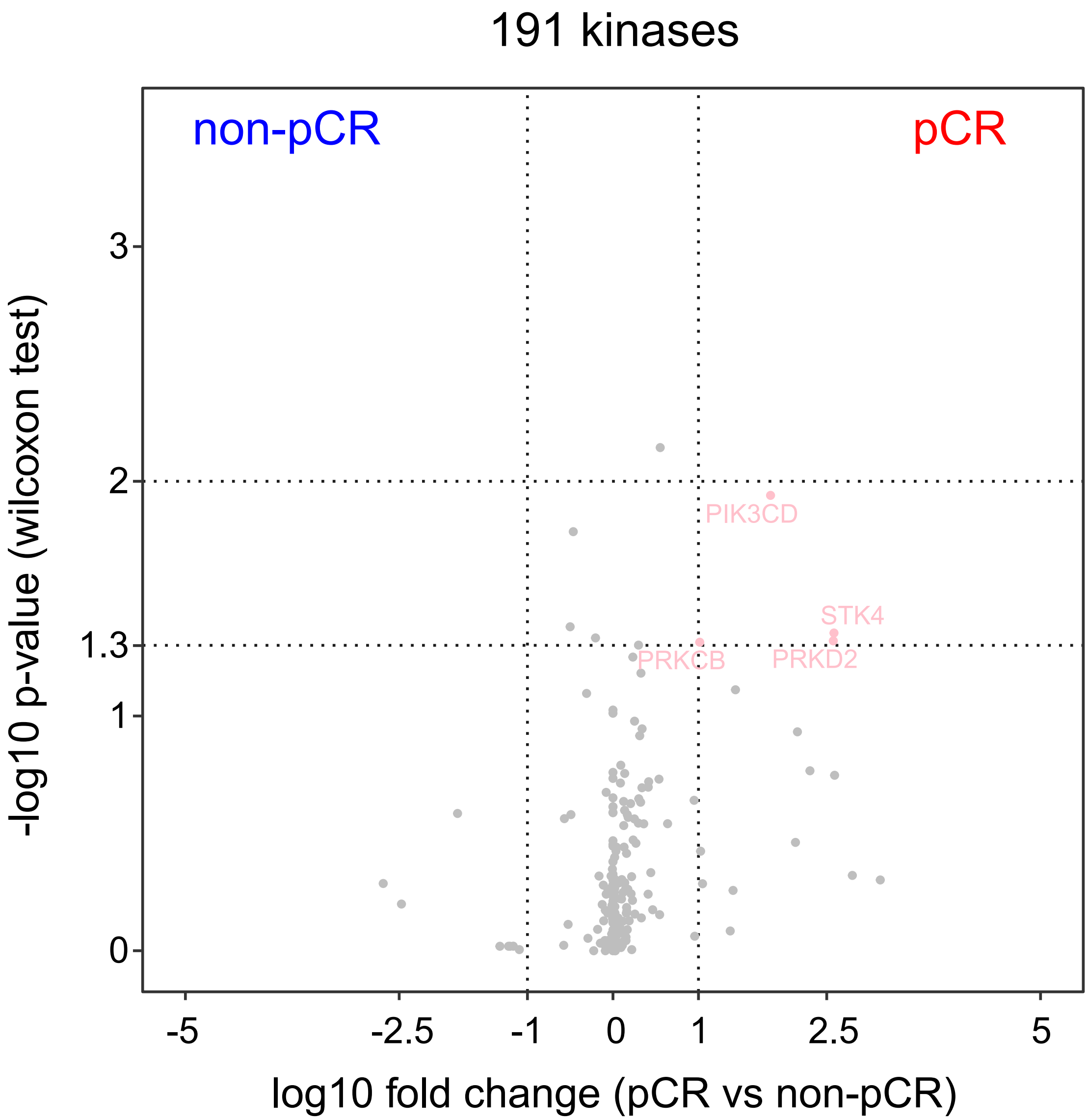

Supplementary Figure 1. (A) Venn diagram shows the overlapping number of proteins across KIPA, RNA-seq and TMT-based proteomics. (B) Volcano plot shows results from differential analysis of human kinases between pCR and non-pCR samples with the KIPA. X-axis is the log10 fold change between median value of pCR and non-pCR samples for each kinase. Y-axis is the -log10 p-value from Wilcoxon rank-sum tests comparing kinase levels in pCR samples to non-pCR samples. Pink points indicate significant ($0.01 < p < 0.05$) proteins.
