# Supplementary material for: Kinase Inhibitor Pulldown Assay Identifies a Chemotherapy Response Signature in Triple-negative Breast Cancer Based on Purine-binding Proteins
Source: Cancer Res Commun. 2023 Aug 15;3(8):1551–63. doi: 10.1158/2767-9764.CRC-22-0501 (PMC10426551; doi:10.1158/2767-9764.CRC-22-0501)
Supplement: Supplementary Figure 2 [file crc-22-0501-s03.pdf]

# Supplementary Figure 2

A

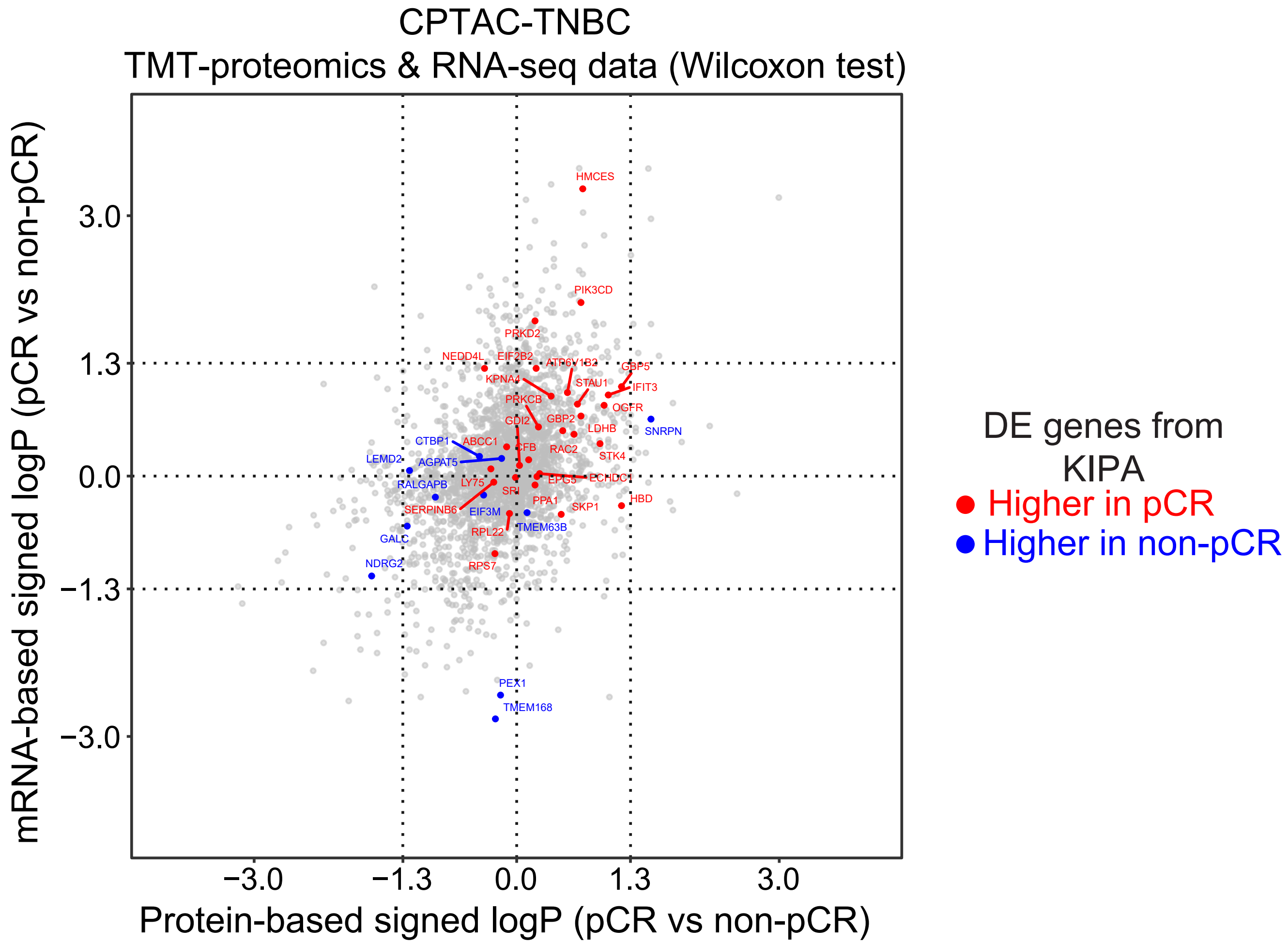


B

| Gene Symbol | Citation number ("chemotherapy" and "breast cancer") |
|---|---|
| GBP2 | 1 |
| GBP5 | 1 |
| RAC2 | 1 |
| NEDD4L | 2 |
| LDHB | 5 |
| KPNA4 | 0 |
| ATP6V1B2 | 0 |

C

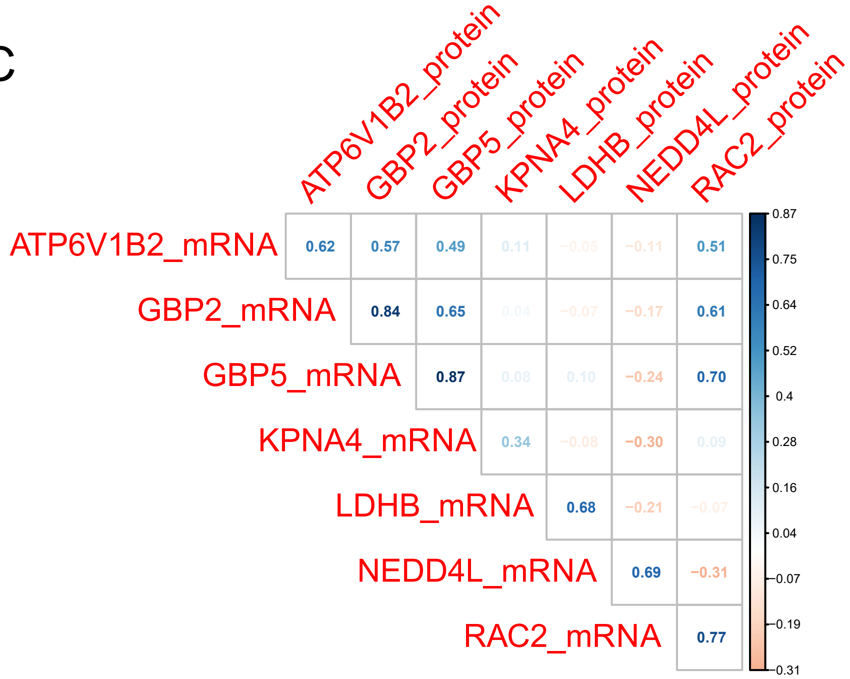


Supplementary Figure 2. (A) Scatter plot shows the signed -log10 p-value of genes (identified by the KIPA) in TMT-based proteomics and RNA-seq of the CPTAC-TNBC study. p-values were determined by Wilcoxon rank-sum tests comparing the protein (x-axis) and mRNA (y-axis) levels for pCR samples to non-pCR samples. Differential genes from the KIPA ($p < 0.05$) were highlighted in red (higher in pCR) or blue (higher in non-pCR). (B) Citation number of each signature protein in the field of "chemotherapy" and "breast cancer" was obtained using R package RISmed. (C) Matrix shows the correlation between the mRNA levels and protein levels of seven PBP signature genes in the CPTAC-TNBC dataset. The number is the correlation coefficient of each spearman correlation. Blue and red represent positive and negative correlation respectively.
