# Supplementary material for: Kinase Inhibitor Pulldown Assay Identifies a Chemotherapy Response Signature in Triple-negative Breast Cancer Based on Purine-binding Proteins
Source: Cancer Res Commun. 2023 Aug 15;3(8):1551–63. doi: 10.1158/2767-9764.CRC-22-0501 (PMC10426551; doi:10.1158/2767-9764.CRC-22-0501)
Supplement: Supplementary Figure 3 [file crc-22-0501-s04.pdf]

Supplementary Figure 3

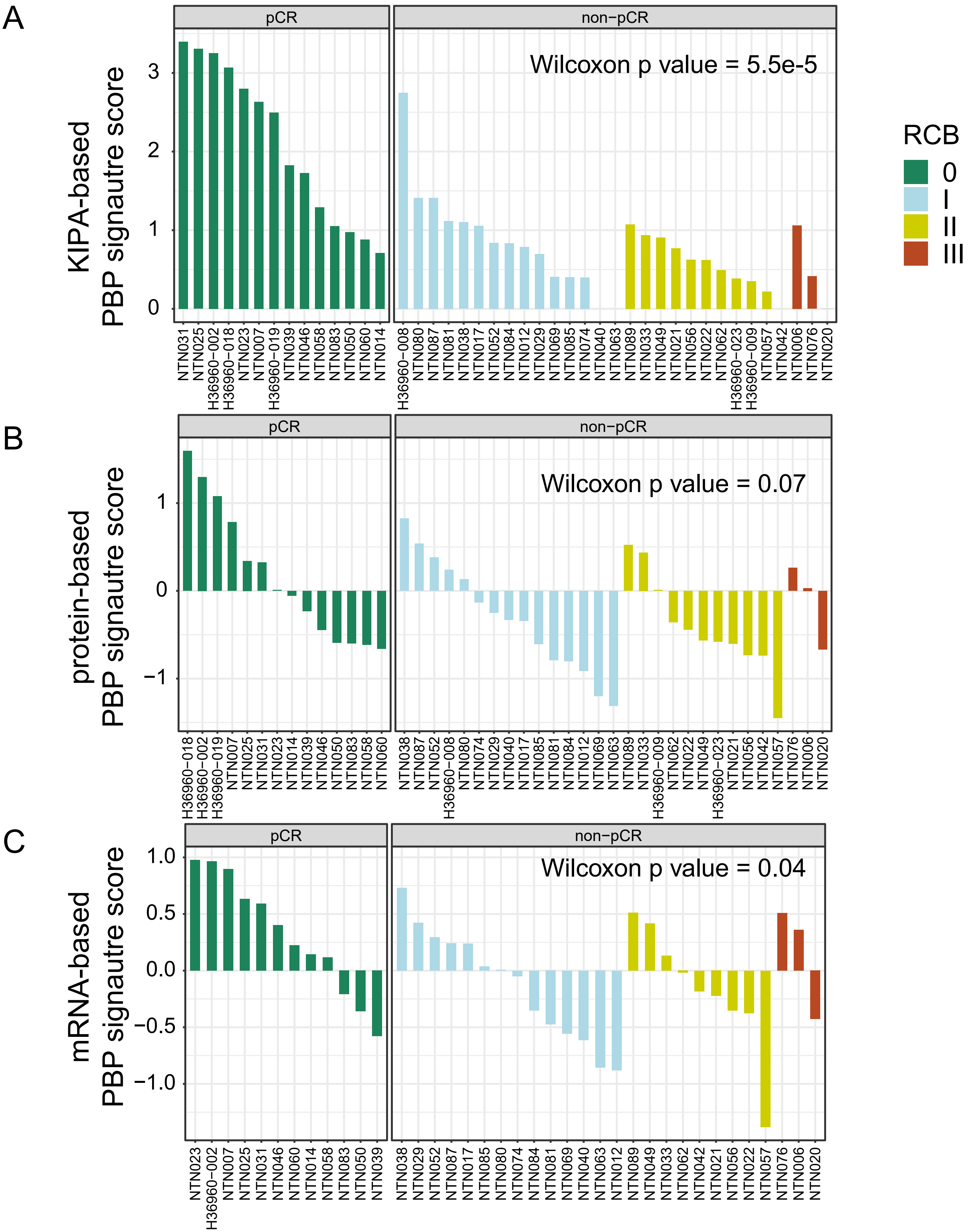

Supplementary Figure 3. Bar plot shows the levels of the PBP signature score in pCR samples (RCB 0) and non-pCR samples (RCB I/II/III) measured by the KIPA (A), the proteomics (B), and the RNA-seq (C). P-values were determined by the Wilcoxon rank-sum test comparing signature levels for pCR samples to non-pCR samples.
