# Supplementary material for: Kinase Inhibitor Pulldown Assay Identifies a Chemotherapy Response Signature in Triple-negative Breast Cancer Based on Purine-binding Proteins
Source: Cancer Res Commun. 2023 Aug 15;3(8):1551–63. doi: 10.1158/2767-9764.CRC-22-0501 (PMC10426551; doi:10.1158/2767-9764.CRC-22-0501)
Supplement: Supplementary Figure 4 [file crc-22-0501-s05.pdf]

Supplementary Figure 4

A Hallmark IFN gamma pathway leading edge genes (protein)

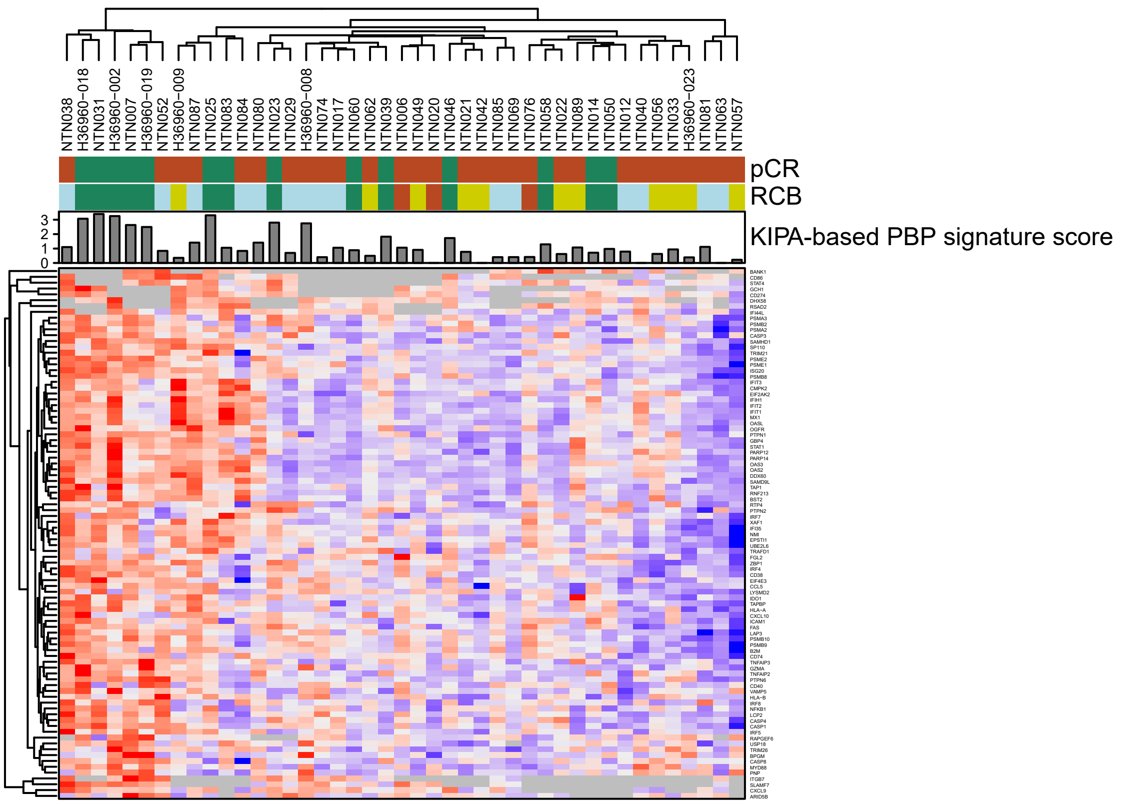

B Hallmark IFN gamma pathway leading edge genes (mRNA)

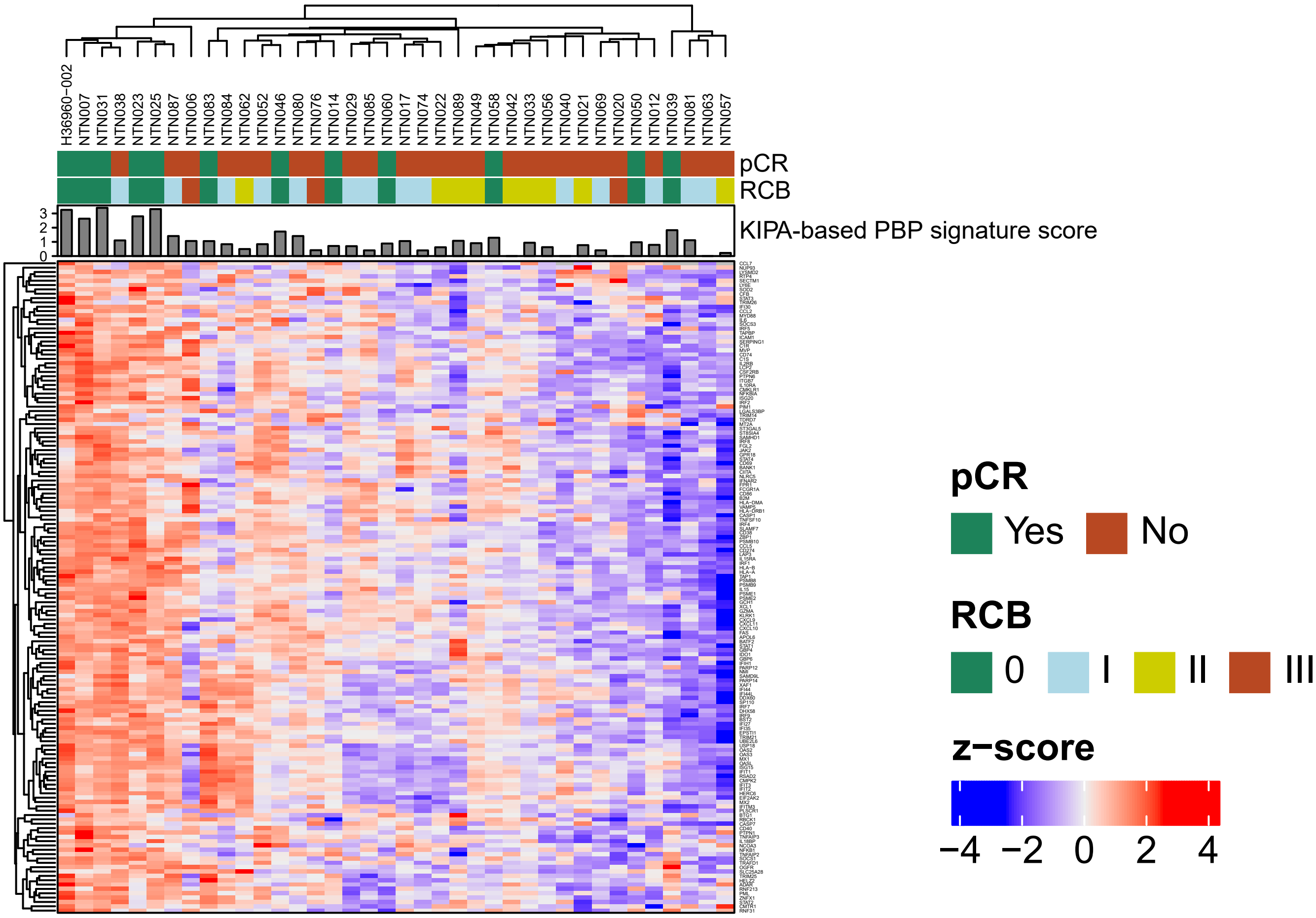

Supplementary Figure 4. Hierarchical clustering heatmap shows the protein (A) and mRNA (B) levels of the leading-edge genes of INTERFERON_GAMMA_RESPONSE Hallmark pathway across samples.
