# Supplementary material for: Kinase Inhibitor Pulldown Assay Identifies a Chemotherapy Response Signature in Triple-negative Breast Cancer Based on Purine-binding Proteins
Source: Cancer Res Commun. 2023 Aug 15;3(8):1551–63. doi: 10.1158/2767-9764.CRC-22-0501 (PMC10426551; doi:10.1158/2767-9764.CRC-22-0501)
Supplement: Supplementary Figure 5 [file crc-22-0501-s06.pdf]

Supplementary Figure 5

A. BrighTNess dataset

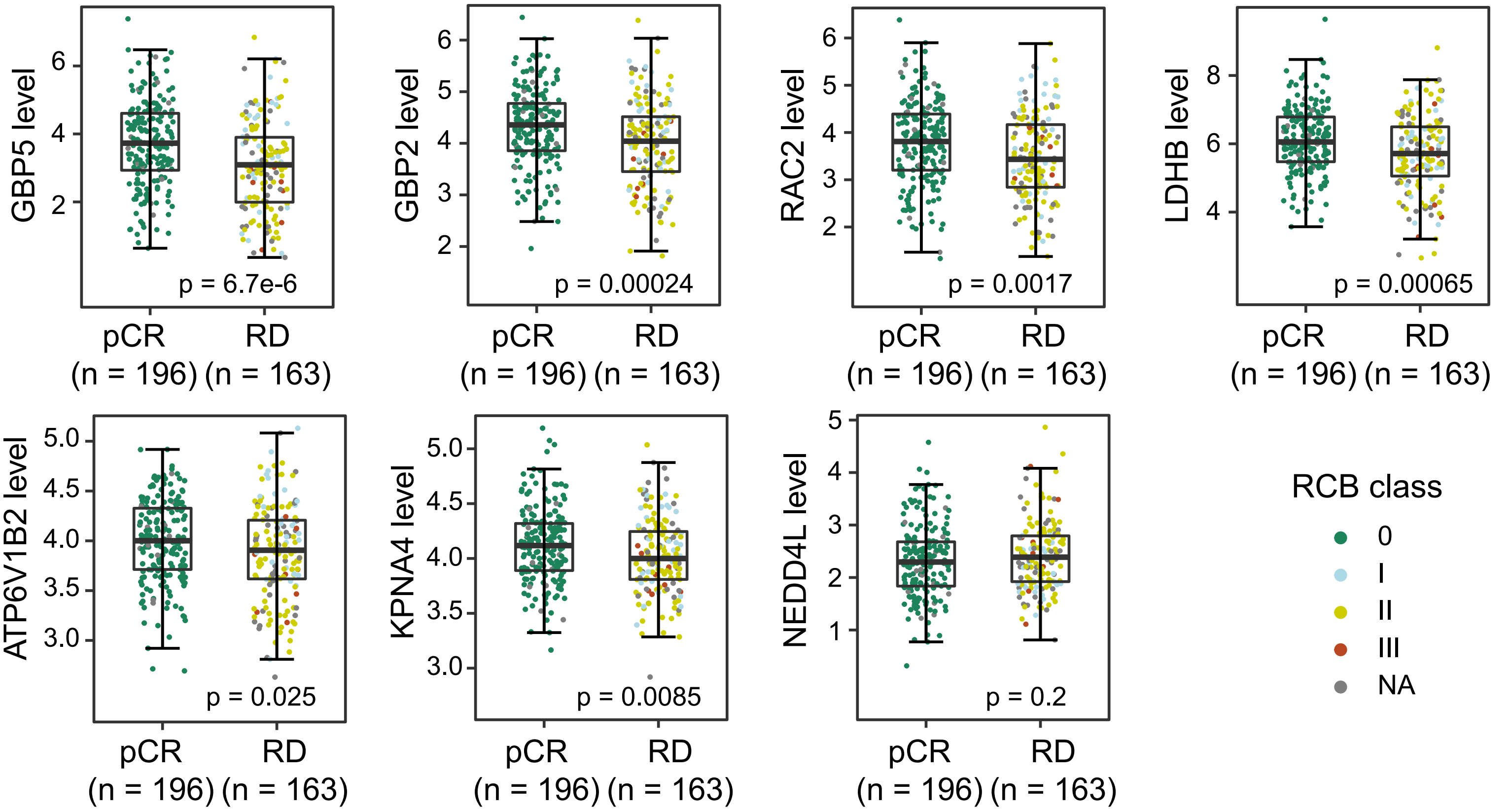

B. GSE18864: TNBC tumors profiled prior to cisplatin

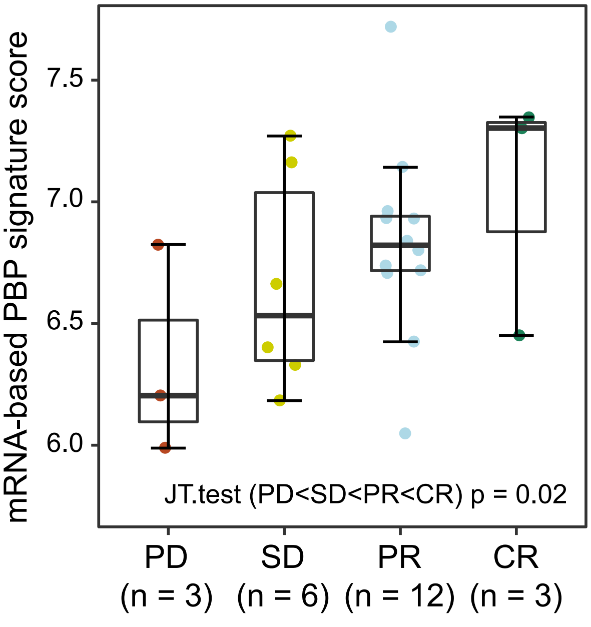

Supplementary Figure 5. (A) The mRNA expression levels of seven PBP signature genes (GBP5, GBP2, RAC2, LDHB, ATP6V1B2, KPNA4, and NEDD4L) in pCR and RD samples of the BrighTNess dataset. Boxplots show interquartile range (IQR) with median marked in center. Whiskers indicate 1.5x IQR. P-values were determined by Wilcoxon rank-sum test between pCR and RD samples. (B) Boxplot shows the mRNA-based PBP signature scores across 24 baseline TNBC tumors receiving cisplatin monotherapy. Response Evaluation Criteria in Solid Tumors (RECIST) as follows: PD, progressive disease; SD, stable disease; PR, partial response; CR, complete response. Data is derived from GSE18864. P-value was determined by Jonckheere-Terpstra test with a rank of PD<SD<PR<CR.
