# Supplementary material for: Kinase Inhibitor Pulldown Assay Identifies a Chemotherapy Response Signature in Triple-negative Breast Cancer Based on Purine-binding Proteins
Source: Cancer Res Commun. 2023 Aug 15;3(8):1551–63. doi: 10.1158/2767-9764.CRC-22-0501 (PMC10426551; doi:10.1158/2767-9764.CRC-22-0501)
Supplement: Supplementary Figure 6 [file crc-22-0501-s07.pdf]

Supplementary Figure 6

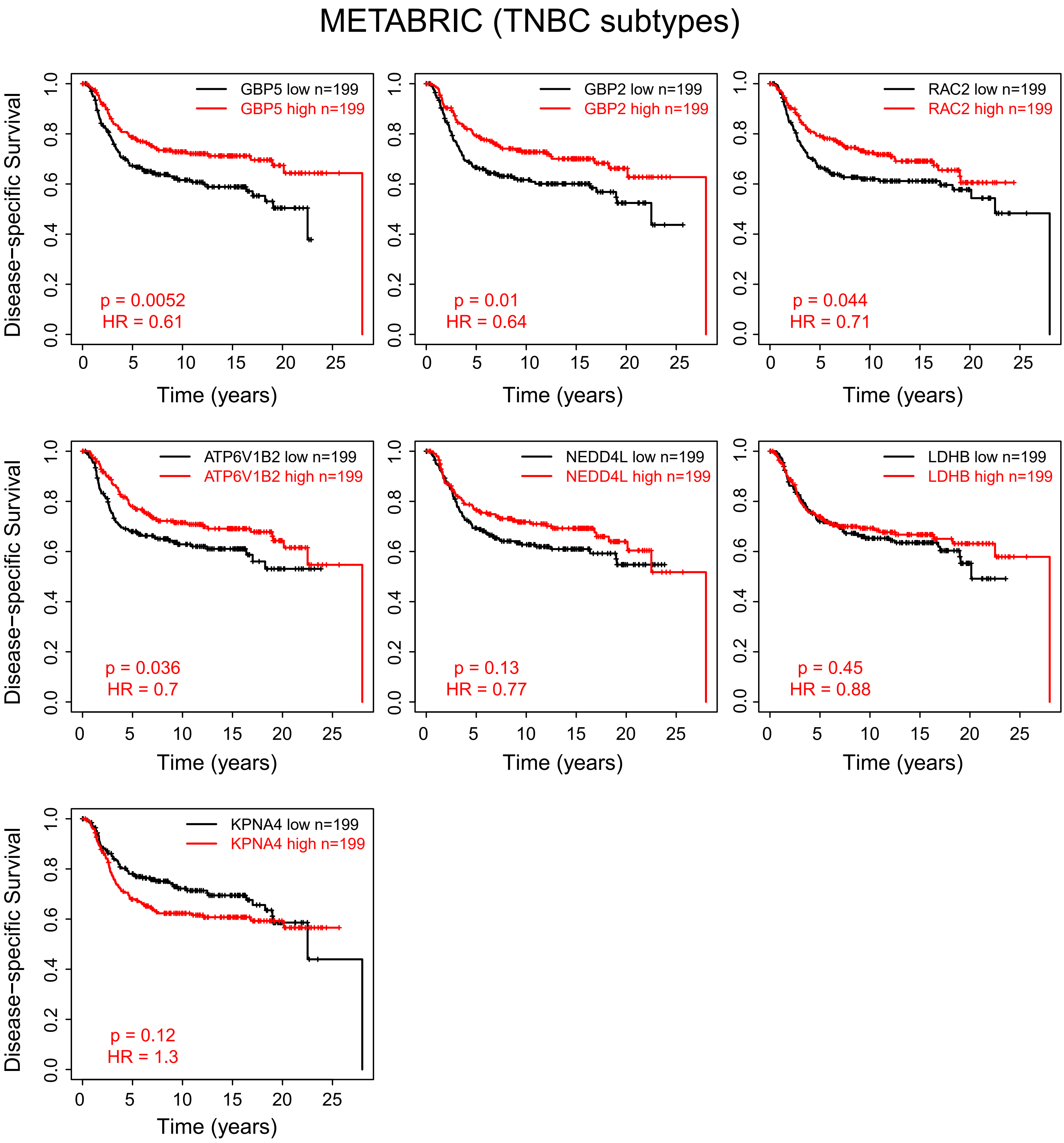

Supplementary Figure 6. Kaplan-Meier (KM) curves show disease-specific survival of TNBC samples from the METABRIC dataset. Samples were categorized into high and low levels of individual signature genes (GBP5, GBP2, RAC2, ATP6V1B2, NEDD4L, LDHB, and KPNA4) based on the median mRNA expression cutoff.
