# Supplementary material for: Kinase Inhibitor Pulldown Assay Identifies a Chemotherapy Response Signature in Triple-negative Breast Cancer Based on Purine-binding Proteins
Source: Cancer Res Commun. 2023 Aug 15;3(8):1551–63. doi: 10.1158/2767-9764.CRC-22-0501 (PMC10426551; doi:10.1158/2767-9764.CRC-22-0501)
Supplement: Supplementary Figure 8 [file crc-22-0501-s09.pdf]

Supplementary Figure 8

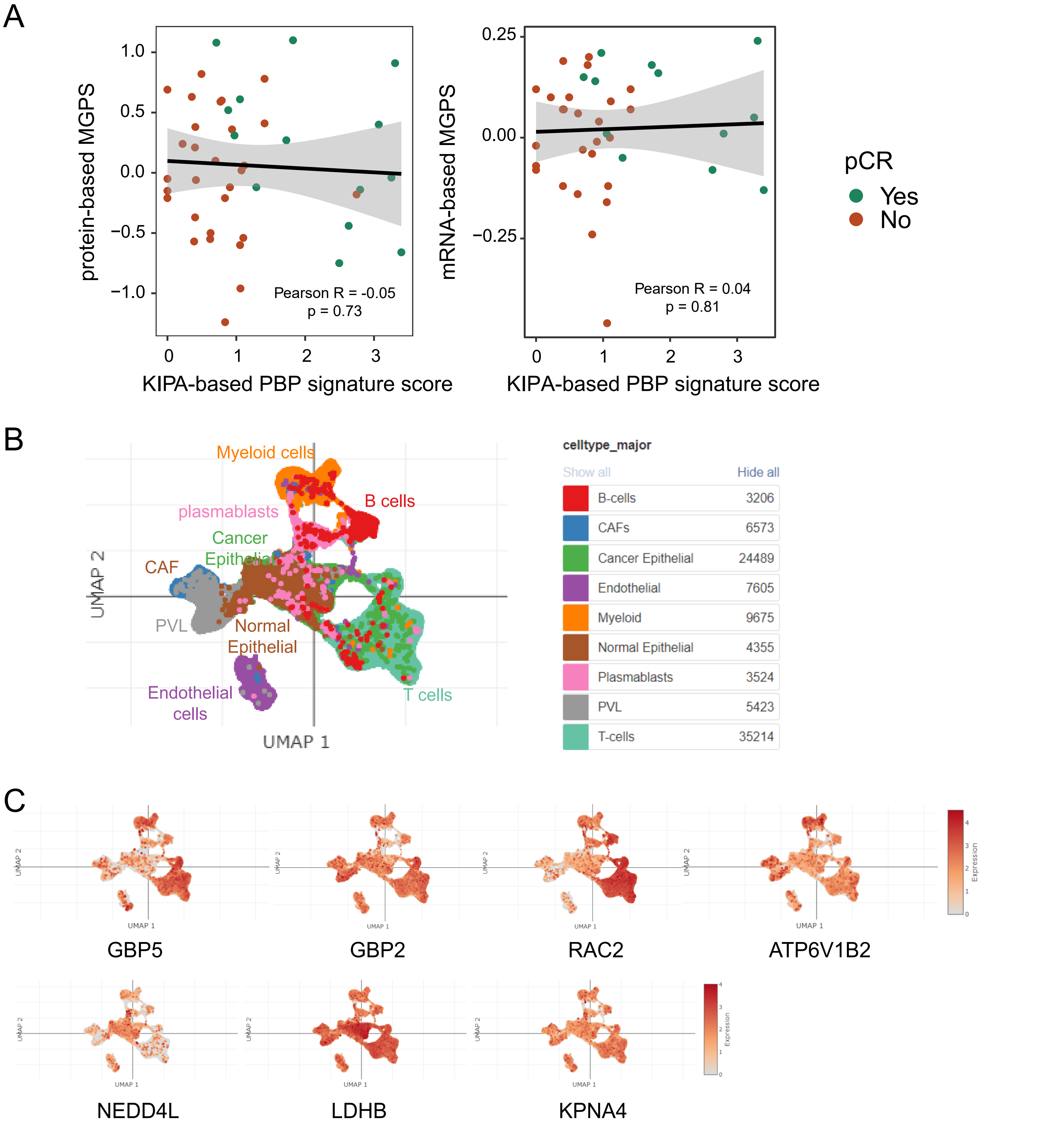

Supplementary Figure 8. (A) Scatter plot shows the correlation between the KIPA-based PBP signature score and protein-based or mRNA-based multigene proliferation scores (MGPS). (B) Single-cell RNA-seq data from a public dataset with 26 primary breast tumors. UMAP visualization shows the cell types annotated in the previous publication (30), generated from the Broad Institute Single Cell portal at https://singlecell.broadinstitute.org/single_cell/study/SCP1039. (C) Log-normalized expression of seven PBP signature genes in different clusters, generated from the Broad Institute Single Cell portal.
